# Capillary Flow-MRI: Quantifying Micron-Scale Cooperativity in Complex Dispersions

**DOI:** 10.1021/acs.analchem.3c01108

**Published:** 2023-10-05

**Authors:** Klaudia W. Milc, Thomas Oerther, Joshua A. Dijksman, John P. M. van Duynhoven, Camilla Terenzi

**Affiliations:** † Laboratory of Biophysics, Wageningen University, 6708 WE Wageningen, The Netherlands; ‡ Bruker BioSpin GmbH, 76275 Ettlingen, Germany; § Physical Chemistry and Soft Matter, Wageningen University, 6708 WE Wageningen, The Netherlands; ∥ Van der Waals-Zeeman Institute, University of Amsterdam, 1098 XH Amsterdam, The Netherlands; ⊥ Unilever Foods Innovation Centre Hive, 6708 WH Wageningen, The Netherlands

## Abstract

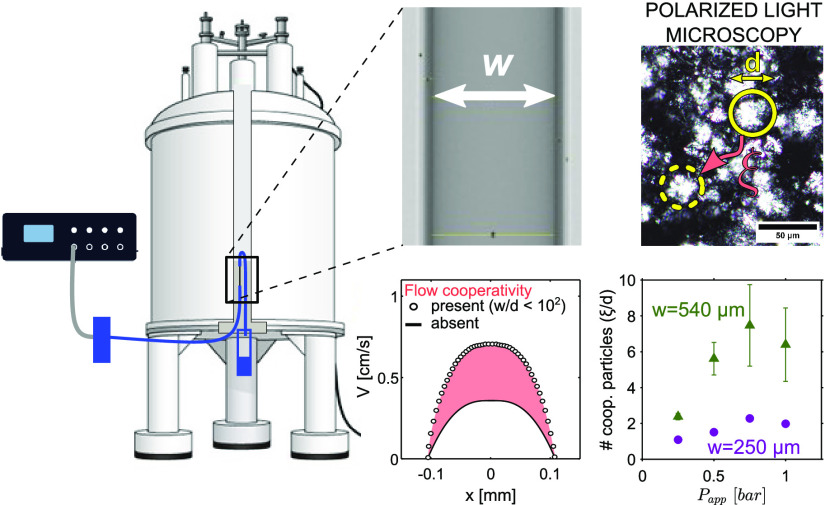

Strongly confined flow of particulate fluids is encountered in applications ranging from three-dimensional (3D) printing to the spreading of foods and cosmetics into thin layers. When flowing in constrictions with gap sizes, *w*, within 10^2^ times the mean size of particles or aggregates, *d*, structured fluids experience enhanced bulk velocities and inhomogeneous viscosities, as a result of so-called cooperative, or nonlocal, particle interactions. Correctly predicting cooperative flow for a wide range of complex fluids requires high-resolution flow imaging modalities applicable *in situ* to even optically opaque fluids. To this goal, we here developed a pressure-driven high-field magnetic resonance imaging (MRI) velocimetry platform, comprising a pressure controller connected to a capillary. Wall properties and diameter could be modified respectively as hydrophobic/hydrophilic, or within *w* ∼ 100–540 μm. By achieving a high spatial resolution of 9 μm, flow cooperativity length scales, ξ, down to 15 μm in Carbopol with *d* ∼ 2 μm could be quantified by means of established physical models with an accuracy of 13%. The same approach was adopted for a heterogeneous fat crystal dispersion (FCD) with *d* and ξ values up to an order of magnitude higher than those for Carbopol. We found that for strongly confined flow of Carbopol in the 100 μm capillary, ξ is independent of flow conditions. For the FCD, ξ increases with gap size and applied pressures over 0.25–1 bar. In both samples, nonlocal interactions span domains up to about 5–8 particles but, at the highest confinement degree explored, ∼8% for FCD, domains of only ∼2 particles contribute to cooperative flow. The developed flow-MRI platform is easily scalable to ultrahigh field MRI conditions for chemically resolved velocimetric measurements of, *e.g.*, complex fluids with anisotropic particles undergoing alignment. Future potential applications of the platform encompass imaging extrusion under confinement during the 3D printing of complex dispersions or in *in vitro* vascular and perfusion studies.

## Introduction

Flow of particulate fluids, such as dispersions or microgels, is encountered in daily life and many industrial processes, with examples including squeezing cosmetics out of packings or flow of foods through pipes and nozzles during their production. In many such cases, flow of particulate fluids is well described and can be successfully predicted by global rheological models, such as Herschel–Bulkley (HB).^1−5^ However, complex fluids tend to exhibit flow instabilities such as wall slip^6^ and shear-history dependence in flow, known as thixotropy,^7^ significantly complicating flow modeling. Additionally, the global rheology approach tends to fail in describing the flow behavior under conditions where the flow confinement size, *w*, is within 2 orders of magnitude of the average microstructural size d, of the flowing fluid. Such conditions can be encountered in food three-dimensional (3D) printing^8,9^ or spreading of cosmetics and paints into thin layers. In such strongly confined regime, where the degree of confinement *d*/*w* ≥ 1%, flow can become cooperative, due to nonlocal interactions between the flowing particles. This typically leads to an increase in the macroscopic velocity and to the onset of spatially heterogeneous viscosity, as compared to nonconfined flow conditions.^10−12^ Thus, accurately predicting strongly confined flow, accounting for cooperativity effects in nonmodel or industrially relevant structured fluids, inherently requires broadly applicable velocimetry techniques with high spatial resolution. Since the first study of flow cooperativity, approximately two decades ago by optical velocimetry measurements, the latter have been used to investigate the dependence of cooperativity on the gap size and on the wall properties of the flow geometry. Recent work also revealed impact of microstructural properties, such as size distribution, concentration, and anisotropy of particles within optically transparent, colloidal model dispersions.^11,13−15^ Problematically, most structured fluids are optically opaque due to the presence of bubbles, droplets, or other particles and thus cannot be measured by optical imaging methods. Under such circumstances, magnetic resonance imaging (MRI) velocimetry can be beneficial, as it does not suffer from this limitation. In recent years, MRI velocimetry has been applied to unravel flow cooperativity in fluids with particle sizes ≳30 μm, like cellulose, fat crystal dispersions (FCDs), and milk microgels, or even granular materials.^16−21^ These studies were performed using rotational rheo-MRI setups^22,23^ equipped with commercially available or custom-made^24^ Couette cells (CC) with gap sizes down to 0.5 mm, or cone–plate (CP) geometries with angles down to 4°. Figure S1 in the Supporting Information (SI) shows the scheme of a rotational rheo-MRI setup with both CC and CP geometries. On the one hand, the cylindrical symmetry of the CC geometry lends itself well to MRI measurements, while on the other hand, the stress distribution is approximately homogeneous in a CP geometry. Yet, both geometries possess their limitations, which impede their use for wide-range studies of cooperativity. Specifically, downsizing the gap within a CC geometry below 0.5 mm^24^ is not realistic, due to challenges in the vertical alignment of the concentric cylinders inside the magnet, and in the mechanical stability of the rotating parts.^24^ Conversely, because of the increasing gap size along the radial direction of a CP geometry, imposing a well-defined degree of flow confinement, increasing the imaging slice thickness to achieve a higher signal-to-noise ratio (SNR), and shearing dense or heterogeneous structured fluids is a challenge. For both of these geometries, modifying wall properties is costly and time-consuming. Furthermore, the CCs and CPs along with the rheo-MRI drive shafts (see Figure S1 in the SI) must be manufactured for specific bore sizes of MRI magnets and are currently not available for narrow-bore and/or ultrahigh field MRI magnets. All these limitations have thus far prevented broadening the scope of high-field flow-MRI measurements toward micrometer-scale cooperativity studies. To bridge this gap, in this work, we have developed a capillary flow-MRI platform for translational flow measurements, available with various capillary sizes and wall properties, and easily scalable for use at any magnetic field strength and magnet bore size. These features of the platform broaden the experimental conditions under which cooperative flow can be studied and also open the possibility to study industrially relevant processes including extrusion^25,26^ and mixing of multiphase fluids in sub-mm confinements.^27^

We first demonstrate the validation of the proposed flow-MRI platform by determining the local viscosities of a Newtonian fluid, namely silicone oil, flowing in capillaries with diameters within 100–540 μm. Subsequently, we study the confined flow of two particulate fluids: Carbopol and FCD as a function of the capillary diameter and wall properties. Carbopol, typically containing particle sizes in the order of 1–10 μm,^3,14,28^ is often used as a model yield stress fluid in rheological studies and, in this work, it enables benchmarking the capability of our approach in estimating cooperativity lengths for a fluid with particle sizes below the spatial MRI resolution. On the other hand, FCDs with crystal aggregate sizes from hundreds nm up to ∼200 μm in diameter^29−32^ are relevant for the processing of foods, such as margarine or chocolate. With the proposed platform, we were able to quantify flow cooperativity over the broad range of particle sizes covered by the two particulate dispersions, with relevance in rheological and industrial applications. We found that in Carbopol, cooperativity appears independent of strongly confined flow conditions with only a minor effect from wall properties, while in the FCDs, with larger particle sizes, the capillary diameter largely affects the cooperativity length scales. In the final part, we demonstrate that higher stress variation within the flow geometry leads to a decrease of ξ values, by comparing the results obtained with the developed capillary setup and a corresponding CC geometry.

## Materials and Methods

### Sample Preparation

As a model Newtonian fluid, silicone oil, with viscosity at 25 °C η_25°_ = 1 Pa·s was purchased from Sigma-Aldrich and used with no further modifications. As a model yield stress fluid, Carbopol was prepared following the procedure described by Géraud et al.^14^ Carbopol ETD 2050 powder (Lubrizol) was added to Milli-Q water heated to 50 °C, in a ratio to give a 0.5 wt % solution, after which the dispersion was stirred for 30 min with a magnetic stirrer at 50 °C until a complete dissolution of the powder. The resulting solution was cooled to room temperature (RT), and the pH was adjusted from ∼3 to 7.0 ± 0.5, by dropwise addition of aqueous NaOH (5 M), under continuous stirring. During the neutralization, jamming of the polymer network occurs due to swelling of the polymer blobs with water.^28^ It is known from the literature that the stirring method affects the microstructure.^14^ Hence, the resulting arrested microgel was stirred with a mixer for 24 h at 2000 rpm at RT to obtain the desired microstructure size of ∼3 μm. After preparation, the sample was stored at RT. For preparation of the 15% FCD, the solid fat blend was mixed with a commercially available sunflower oil (SF) to give a 15 wt % fat-in-oil dispersion, which was heated at 50 °C for 20 min to erase crystal polymorphic history. Subsequently, the melt was transferred to a vessel preset at 15 °C for isothermal crystallization over 10 min. A fresh batch of sample was prepared before each experiment and used within 8 h from preparation.

### Microstructure Determination

The microstructure of Carbopol was visualized under a confocal microscope (Nikon Ti2 Eclipse), equipped with a high sensitivity camera (Nikon C2). A drop of Carbopol, stained with rhodamine B, was deposited on a glass slide and examined with an oil objective (60× magnification, NA = 1.4). A laser with a wavelength of λ = 561 nm was used for excitation with the collected fluorescence bandwidth set to 510–593 nm. The microstructure of the FCD was visualized using a polarized light microscope (Nikon Eclipse) equipped with a 10× objective lens. The images were acquired with an Olympus DP70 camera and digitalized using cellSens imaging software. The mean sizes of the polymer blobs in Carbopol and of the crystal aggregates in the FCD sample were obtained from the intensity correlation spectroscopy approach, as described in detail by Géraud et al.^14^ The two-dimensional (2D) autocorrelation function of the micrographs of both samples was calculated and radially averaged. The resulting exponential decay was fitted with the following equation
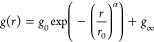
1where *r*_0_ is the correlation length, an equivalent of particle radius, *g*_0_ is the peak intensity, and *g*_∞_ is the background intensity.

### μCT Measurements

The glass capillaries were fitted on the rotation table of the μCT and imaged using a Skyscan 1172 desktop μCT system with a 100 kV X-ray source (10 W, 20–100 kV, 0–250 μA, <5 μm spot size) and a 11 Mp X-ray detector (4000 × 2664 pixels). A power setting of 60 kV and 167 μA was used. Images were acquired using a step size of 0.3° over 360° with a camera binning of 4 × 4 and frame averaging of 2. The pixel size was set to 3.75 μm. The total scanning time, yielding 1200 projection images, was just under 1 h. A stack of 631 horizontal cross sections of 292 × 292 pixels was obtained after tomographic reconstruction of a small region of interest of the projection images. A beam hardening correction of 50% and ring artifact correction of 100 and smoothing of 2 were selected. Avizo V2022.1 was used to generate ortho-slices, and with the ruler function, it was possible to determine the internal diameter.

### Flow-MRI Platform Design

**Figure 1:**
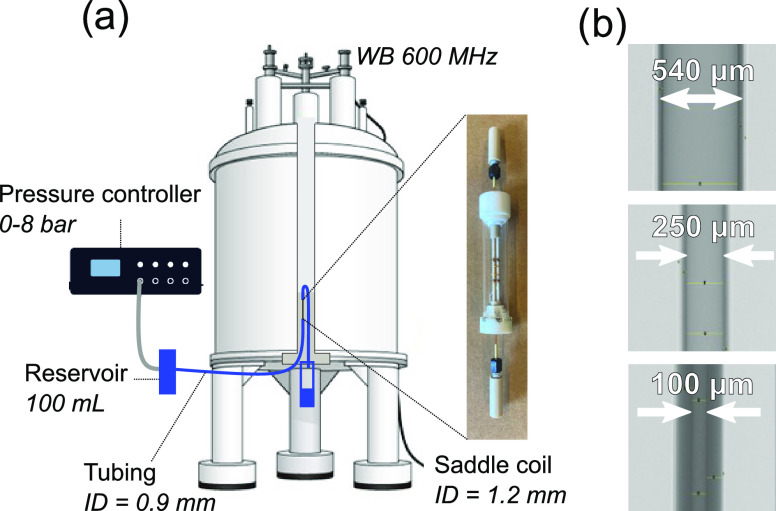
(a) Scheme of the capillary flow-MRI platform in a WB 600 MHz NMR spectrometer. (b) μCT images of the glass capillaries, used for quantification of the capillary inner diameters.

The capillary flow-MRI platform is schematically shown in Figure 1. The components of the platform were purchased from commercial suppliers, namely: (1) the pressure controller (Elveflow) for controlling the applied pressure, *P*_app_ in the range 0–8 bar with an operational error of ±1 mbar; (2) the PTFE connecting tubing with *w* = 0.9 mm (BGB Analytik); (3) the hydrophilic and hydrophobic glass capillaries with diameters in the range 100–540 μm (BGB Analytik); and (4) the PEEK connectors with *w* = 0.5 mm (BGB Analytik). Since velocities in cylindrical capillaries scale according to (*w*/2)^2^, we have measured the radius of all capillaries with μCT (see Figure S2 in the SI). The 1.2 mm saddle coil was supplied by Bruker BioSpin and was adapted in-house. For all experiments, the length of the capillary *L* = 14.7 ± 0.2 cm. The fluid of interest is placed in the reservoir, and the target pressure is applied. The sample flows in the tubing and through the connected glass capillary positioned inside the saddle coil. If the sample exhibits spatial heterogeneities, *e.g.*, due to sedimentation or aging, it is possible to place the reservoir on a stirrer plate and to apply continuous stirring with a magnetic bar during the course of the measurement. Such an approach was followed here for the FCD. The flow measurements were carried out at a constant temperature of 20 °C, which is set by the temperature of the cooling water of the triple axis MRI gradients.

### Flow-MRI Measurements

The ^1^H MRI velocimetry measurements were performed on a wide-bore Avance NEO Bruker spectrometer operating at 14 T. Excitation and detection of the ^1^H signal was performed with a saddle radiofrequency coil with an inner diameter of 1.2 mm, in a Micro 5 microimaging gradient system (Bruker BioSpin) with the maximum gradient intensity of 3 T m^–1^ along all three directions.

2D ^1^H MRI velocity maps were measured using a Pulsed Gradient Spin Echo (PGSE) sequence,^22^ within a 2 mm thick slice in the flow direction, echo time *T*_E_ = 10 ms and repetition time *T*_R_ = 2 s. The duration of the velocity-encoding gradient pulses and their interpulse spacing were δ = 1 ms and Δ = 5 ms, respectively. While for silicone oil and Carbopol we observed only one ^1^H NMR signal from methylene and water protons, respectively, SF oil in FCDs yields ^1^H NMR signals at distinct chemical shifts at the field strength used in this work. Thus, to avoid chemical shift artifacts, a 90° chemically selective, low-power, saturation pulse was used in all velocimetry measurements of FCDs with a bandwidth of 1 kHz and frequency offset of 2.46 kHz.^2^ The field-of-view (FOV) in the read and phase directions was (i) 0.9 × 0.9 mm^2^ for the 540 μm capillary and (ii) 0.6 × 0.6 mm^2^ for both 250 and 100 μm capillaries. By correspondingly acquiring 64 × 64 pixels, isotropic spatial resolutions of 14 and 9 μm could be obtained for measurements (i) and (ii). Using a number of scans NS = 2, the total experiment time per velocity map was 4 min, and three consecutive velocity maps were acquired per sample and *P*_app_ value. Two replicates were acquired for each set of measurements using a fresh sample batch for each repetition. For the data analysis, the 2D velocity maps were radially averaged to yield one-dimensional (1D) velocity profiles. The averaging was performed in MATLAB, with the pixel values binned based on the distance to the center of the capillary. The number of bins was set to 64. As a result, the spatial resolution of the 1D profiles is twice that of the original 2D velocity maps.

### Theoretical Flow Profiles

The theoretical velocities for the Newtonian fluid were calculated from the Hagen–Poiseuille (HP) equation
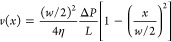
2where Δ*P* is the measured pressure drop between the inlet and outlet of the capillary, and *x* is the position across the gap. For the particulate yield stress fluids, the HB^1^ model was used
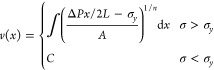
3where σ_*y*_ denotes the yield stress, *A* is a proportionality constant, and *n* is a power law index, all obtained from global rheological measurements. For stresses below the yield stress, σ < σ_*y*_, the velocity is uniform in the center of the capillary and denoted by *C*. We note that for all investigated capillary diameters, the relative stress variation across the radial direction is 100%, with maximum stress at the walls and null value in the center of the capillary. The pressure drop value, Δ*P*, across the capillary length was measured in a separate experiment using a setup located outside the spectrometer, identical to that used for flow-MRI measurements. The pressure sensors were connected directly to the 540 μm capillary, while intermediate tubing with *w* = 0.9 mm was used between the sensor and the 250 or 100 μm capillaries. We note that unlike for the latter capillaries, using a connector tubing with the 540 μm capillary contributed significantly to the measured Δ*P* values, due to the similar inner diameters of both the tubing and the capillary.

### Quantification of Flow Cooperativity

A number of models have been developed to quantify nonlocal effects in particulate materials.^10,11,33,34^ In this work, we use the fluidity model developed by Goyon et al.^12^ for soft, particulate, fluids with rheological properties similar to Carbopol and FCDs. The model is based on the concept of fluidity, defined as the ratio of the shear rate and the shear stress, *f* = γ̇/σ, and is linked, on a microscopic level, to the local rate of plastic rearrangements across the whole flowing system. In the case of strongly confined flow, where the ratio of the mean particle size to confinement size *d*/*w* ≥ 1%, the local fluidity, *f*(*x*), is found to deviate from its predicted global value, *f*_bulk_, and to obey the nonlocal equation^12^
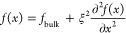
4where ξ is the cooperativity length, and *f*_bulk_ is determined by independent rheological measurements in a nonconfined flow regime. Detailed description of solving and fitting the model to complex fluids flowing through geometries with nonuniform stress fields, such as the microcapillaries used in this work, can be found in our previous work.^20^ Values of ξ for each *P*_app_ can be obtained either by fitting the mean velocity profiles or by fitting profiles from individual measurements and averaging thereafter. For Carbopol, both tested approaches yielded equal results, and here, we report only the results from the former. For the FCD, due to higher variability in velocities, the latter approach was used.

### Rotational Rheology

All rotational rheometric measurements were performed by using an Anton Paar (MCR 301) rheometer. Global flow curves were acquired by using a geometry with a gap size at least 100 times larger than the determined microstructure size of the sample. For Carbopol, a standard stainless-steel CP geometry with a diameter of 25 mm was used. In this geometry, the cone is truncated, with the resulting minimum gap of ∼50 μm in the center. Since the measured stress originates primarily from the outer regions of the CP, where the gap size is ∼200 μm, for Carbopol the wide-gap approximation is fulfilled. The FCD was measured with a standard stainless-steel CC with the radius of the bob *r*_i_ = 5.25 mm and the radius of the cup *r*_o_ = 8.75 mm. Sandpaper was glued to the surface of the bob to prevent slip. Both samples were presheared for 3 min at 30 s^–1^ and, thereafter, exposed to stress measurements under a shear rate sweep from 100 to 0.01 s^–1^. A total of 20 points per decade in shear rate were recorded, averaging each stress value over 5 s.

## Results and Discussion

### Precision of Capillary Flow Measurements

**Figure 2:**
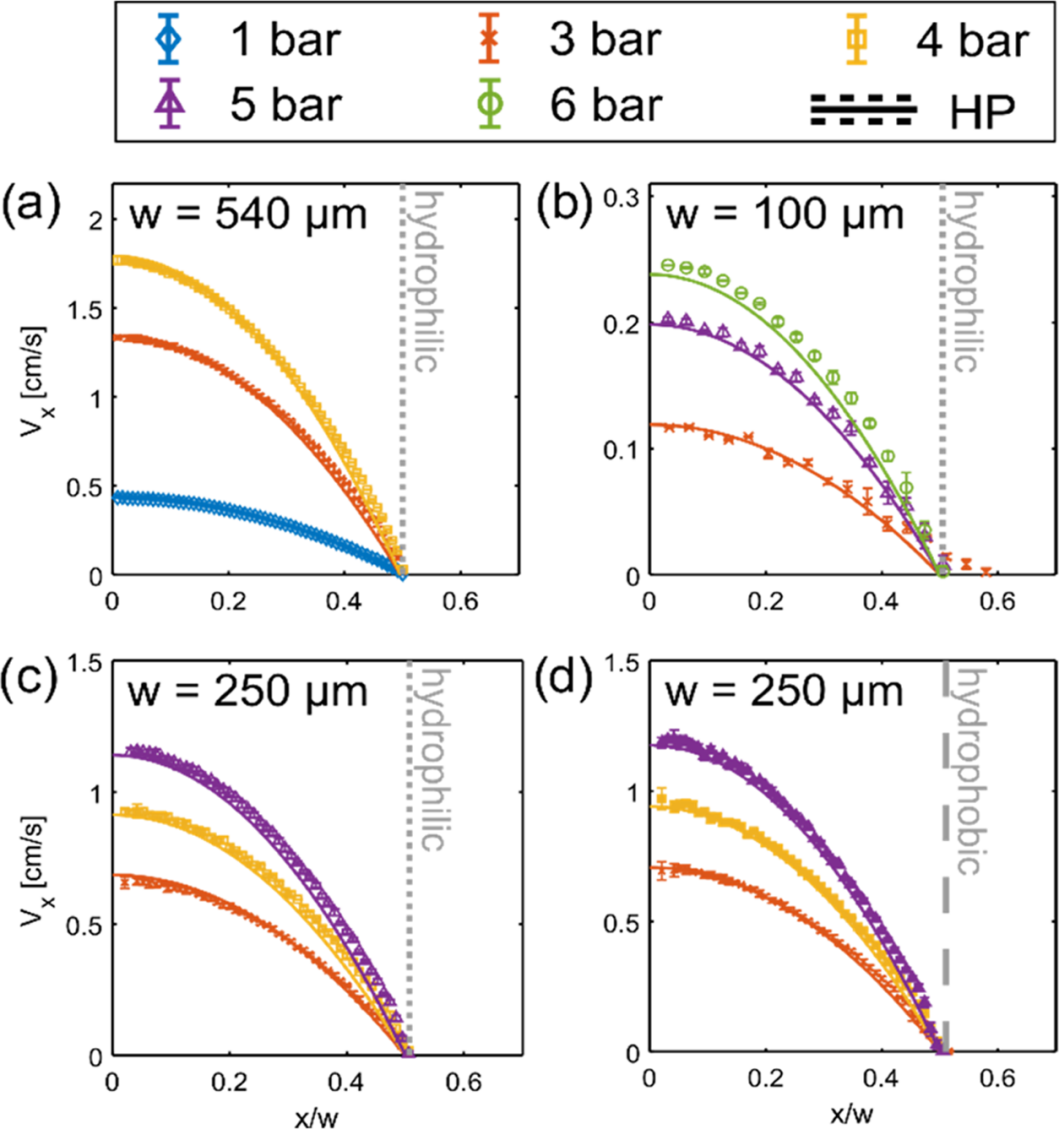
^1^H MRI velocity profiles of silicone oil (η_20°_ = 1.1 Pa·s) acquired at various applied pressures in capillaries with either hydrophilic walls (gray dotted line) and *w* equal to (a) 540 μm, (b) 100 μm, (c) 250 μm, or (d) hydrophobic walls (gray dashed line) and *w* = 250 μm. The error bars represent variation in velocities over 3 time points and 2 replicates. Experimental profiles are compared with theoretical velocities (solid lines) calculated from the HP model (eq 2), plotted against a normalized spatial coordinate *x*/*w*. The uncertainty in theoretical calculations caused by variations in Δ*P* is within the line thickness for all measurements.

We validated the precision of the MRI velocimetry measurements in capillaries with *w* = 100, 250, and 540 μm, using silicone oil with viscosity η_20°_ = 1.1 Pa·s. For the 250 μm capillary, we also tested the effect of wall slip in untreated hydrophilic walls and treated hydrophobic walls. The resulting velocity profiles measured at *P*_app_ = 1–6 bar are shown in Figure 2. All measured flow profiles for silicone oil are well described by the HP equation within the 5% maximum relative deviation. Wall slip is absent in all measurements, as visible by the comparison of data in Figure 2c,d. We also calculated the local flow curves, reporting the local shear rate, γ̇(*x*), obtained from the slopes, *∂V*_*x*_/∂*x*, of all measured velocity profiles, vs the local stress calculated as σ(*x*) = Δ*Px*/2*L*. All the resulting flow curves collapse onto one master curve and are in agreement with separate rheological measurements of viscosity (see Figure S3 in the SI). These results evidence that flow in our setup is temporally stable and reproducible.

### Proof of Concept: Confined Flow of Carbopol

**Figure 3:**
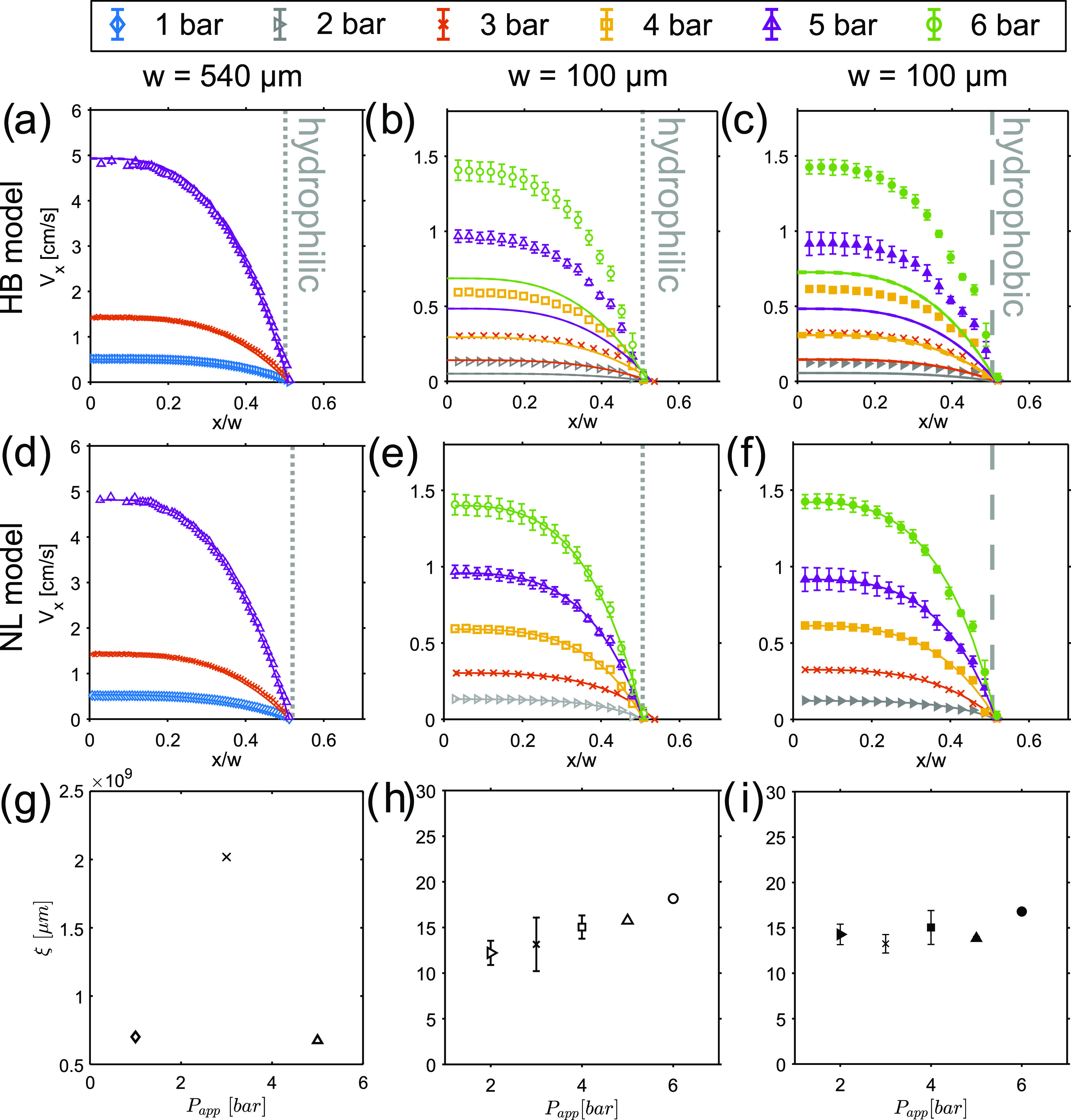
^1^H MRI velocity profiles of 0.5% Carbopol acquired at various *P*_app_ in capillaries with *w* = 540 μm (a, d), 100 μm (b, e), and 100 μm (c, f) with the vertical gray dotted or dashed lines representing, respectively, hydrophilic or hydrophobic walls of the capillaries. The error bars refer to three consecutive measurements of two sample replicates. Experimental velocity profiles are compared with theoretical velocities calculated from the global HB model (solid line, top row) or fitted with the fluidity model (solid line, middle row). The fitted ξ values (h, i) are reported with the fitting error. The fitting of velocity profiles at *w* = 540 μm failed to converge at a solution, resulting in unreliable ξ values, where the error could not be computed (g).

In this section, we study the flow of Carbopol in capillaries with diameters of 540 and 100 μm. In Carbopol, the polymer blob size was determined to be (2.30 ± 0.02) μm (see Figure S4a,b and Table S1 in the SI). Hence, only for the smallest capillary flow of Carbopol is expected to fall within the strongly confined regime. The resulting ^1^H MRI velocity profiles from all above capillaries, with either hydrophilic or hydrophobic walls, are presented in Figure 3a–c. In all measured velocity profiles, a region of nonsheared fluid is detected in the center of the capillary. The coexistence of sheared and nonsheared regions is typical for yield stress fluids and occurs when the applied stress is below the yield stress of the material. In the top row of Figure 3, the experimental velocity profiles are compared with velocities calculated from the global flow behavior measured by conventional rotational CP rheology (Figure S5a in the SI). As expected, only the velocities in the largest capillary, where flow of Carbopol is outside the strong confinement regime, can be correctly predicted by the global HB model. In the smallest capillary, the measured velocities significantly exceed those predicted by the HB model. In the latter condition, no appreciable wall slip effect is observed. By plotting the local flow curves derived from the velocity profiles and the respective Δ*P* measurements (see Figure S6 in the SI), it is evident that flow of Carbopol at *w* = 100 μm cannot be described by a global law and that the flow behavior at each *P*_app_ is unique and exhibits nonlocal character, as a result of cooperativity. As shown in Figure 3d–i, by fitting the mean experimental velocity profiles for Carbopol acquired for *w* = 100 μm with the fluidity model (eq 4), the cooperativity length, denoted as ξ,^12^ could be determined. All experimental velocity profiles appear well described by the fluidity model, although, as expected, for the largest capillary, the fitting subroutine failed to converge towards a reliable solution, which resulted in exceedingly high ξ values (Figure 3g) with no physical meaning. Correspondingly, no reliable estimation of errors could be obtained. For the *w* = 100 μm capillaries, with either smooth or hydrophobic walls, the resulting mean ξ value is 15 ± 2 μm and, as expected, in the order of a few particles. We note that this result is in good agreement with the cooperativity length determined in an identical sample of Carbopol by Géraud et al.,^14^ with a small deviation of 10% possibly originating from differences in the shape and size of the flow geometry.

We note that all the ξ values obtained for Carbopol are on the order of our MRI spatial resolution and thus could be reliably quantified thanks to the high mechanical stability of our platform and high SNR and filling factor achieved with the adapted RF saddle coil This in turn enabled adjusting the FOV to the size of the capillary. Finally, contrarily to what was found for emulsions,^15^ we conclude here that altering the chemical properties of the walls has no significant effect on the interparticle interactions in the flow of Carbopol. Paredes et al.^15^ found that oil droplets in an emulsion adhere to hydrophobic walls of rectangular microchannels, affecting the flow at boundaries. In the case of Carbopol, such adhesion likely does not occur because the continuous and dispersed phases have similar hydrophobic interactions.

### Proof of Concept: Confined Flow of FCD

**Figure 4:**
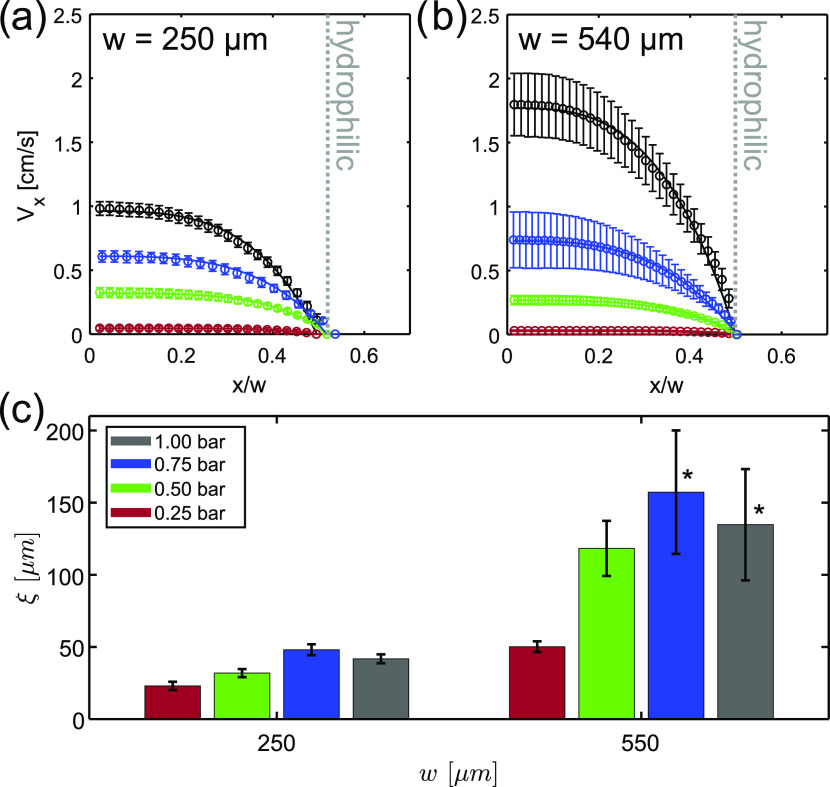
^1^H MRI velocity profiles of 15% FCD acquired at various *P*_app_ in capillaries with *w* equal to (a) 250 μm and (b) 540 μm, plotted as a function of the normalized position, *x*/*w*. The error bars represent variation in velocities between six profiles (3 time points, 2 replicates). Velocity profiles are fitted with the fluidity model (solid lines). Resulting ξ values are shown in (c) for both capillaries with the error bar representing the standard deviation of individual values. All ξ values were calculated from six experiments with the exception of values with an asterisk, where only three experiments were used for calculation.

In our previous work^20^ based on rheo-MRI measurements in a custom-made 500 μm CC, we have shown that flow cooperativity in concentrated FCDs, with 27% solid fat content (SFC), depends on the cooling rate used for sample preparation, as this in turn influences the fat crystal platelet aggregate size and the interaggregate weak-link network.^20^ With the capillary flow-MRI platform proposed here, we can investigate cooperativity at even higher confinement degrees. To this aim, we have measured the flow behavior of a FCD sample, with SFC ∼ 15%, in 540 and 250 μm capillaries, and obtained the mean velocity profiles shown in Figure 4a,b. We note that the oil in FCDs yields ^1^H NMR signals at distinct chemical shifts at the field strength used in this work. With our chosen measurement approach,^2^ chemical shift artifacts were however adequately suppressed. We found that flow is stable over time and reproducible over the two replicates in the 250 μm capillary. In the largest capillary, velocities are temporally stable at all pressures but reproducible across replicates only for the lowest *P*_app_ values, namely, 0.25 and 0.5 bar. At the two highest pressures, the reproducibility error increased up to about 14%. However, velocity enhancement of about 40% (data not shown) with respect to theoretical velocities calculated from the global flow behavior (see Figure S5b in the SI) is observed for both capillaries and at all *P*_app_ values. As established from the autocorrelation function of the micrographs of the sample (see Figure S4c,d in the SI), the mean size of the crystal aggregates in the 15% FCD is (21.06 ± 0.02) μm. It therefore follows that even in the largest capillary, the flow falls within the strongly confined flow regime, with *d*/*w* ≈ 4%. We note that during our velocimetry measurements, we did not detect any particle migration perpendicularly to the flow direction, as evidenced from the uniform ^1^H NMR intensities over the 2D density profiles (see Figure S8 in the SI). With an attempt to model the flow of the FCD in both capillaries and quantify the extent of cooperativity, we fitted the experimental velocity profiles with the fluidity model (eq 4), and the obtained results are presented in Figure 4. Because of the aforementioned variability across the individual profiles, we fitted each profile separately, and in Figure 4c, we report the averaged ξ values. We observe that (i) at each pressure ξ increases by a factor of 2–3 between *w* = 250 μm and *w* = 540 μm and that (ii) ξ reaches the highest values at 0.75 bar for both capillaries. We note that the fluidity model describes well all measured flow profiles at *w* = 250 μm but only those at the two lowest pressures for *w* = 540 μm. For *P*_app_ = 0.75 and 1 bar, ξ values could be reliably quantified only for one of the replicates (see caption of Figure 4). The latter evidence confirms the lower reproducibility of flow measurements at those low pressures. Hence, we conclude that at high applied stresses and relatively low confinement degree of *d*/*w* ≈ 4%, the system exits the cooperative flow regime, and thus, quantification of ξ values is less robust. This is further confirmed by the collapse of the local flow curves onto a master curve at high pressures for the largest capillary (see Figure S7a in the SI).^11^

### Cooperativity Lengths and Microstructure

**Figure 5:**
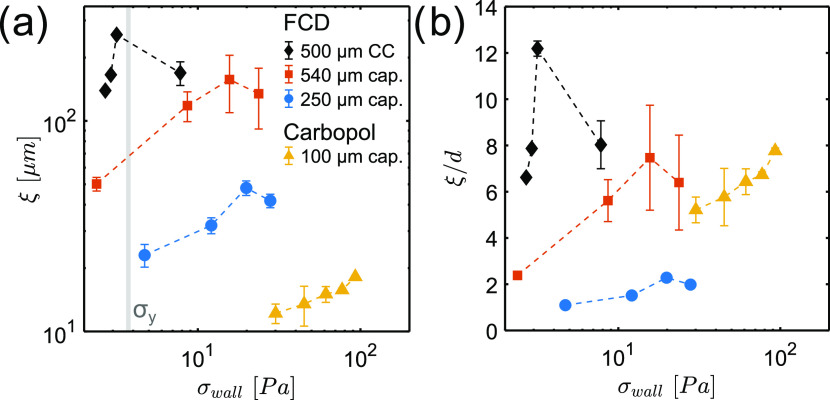
Cooperativity lengths ξ found in flow of FCD and Carbopol in all studied geometries, shown as (a) non-normalized values or (b) values normalized by the mean sizes of the particles, plotted against stress at the wall, calculated from the stress distribution in each geometry. The vertical gray panel in (a) marks the yield stresses of Carbopol and FCD, respectively, equal to 4.07 and 3.46 Pa. The data points are connected with dashed lines as a guide for the eye.

In Figure 5, we compare the ξ values determined for 0.5% Carbopol in the 100 μm capillary and for 15% FCD in the 250 and 540 μm capillaries. For the latter sample, we also include ξ values previously obtained with a rotational rheo-MRI setup using a CC geometry with 500 μm gap size.^24^ Details of rotational rheo-MRI measurements and data analysis are reported in our previous study.^20^ In this rotational setup, the flow is shear-controlled, while in the capillary flow-MRI setup, it is pressure-controlled. For data comparison, we plotted the ξ values against the walls’ stress, σ_wall_, calculated from either the measured Δ*P*, for capillaries, or from the measured torque, for the CC. As can be seen from Figure 5a, ξ values spanning 2 orders of magnitude could be quantified. For Carbopol, ξ varies by only 30% in the pressure range 2–6 bar, while for the FCD, it respectively varies by 50 and 70% at *w* = 250 and 540 μm in the pressure range 0.25–1 bar. This different behavior of Carbopol and FCD agrees with the predictions of the kinetic elasto-plastic (KEP) model,^10^ according to which ξ diverges at the yield stress of the material. As shown in Figure 5a, for Carbopol, we probed cooperativity at σ ≫ σ_*y*_, while for the FCD, measurements were conducted at σ ∼ σ_*y*_. By using either the 500 μm gap CC at γ̇_app_ = 0.5–10 s^–1^ or the 540 μm capillary at *P*_app_ = 0.25–1 bar, consistent ξ values and trends were observed for the FCD sample. We note that ξ values in the 540 μm capillary, with 100% stress inhomogeneity, are ∼35% smaller than those in the CC geometry, where stress inhomogeneity drops to ∼11%. Since the geometry-dependent stress distribution is accounted for in the adopted model, we conclude that the observed difference in ξ arises from a stress-dependent flow cooperativity, as predicted by the KEP model,^10^ and as also seen in studies of cellulose dispersions or soft colloidal microgels.^35^ Finally, in Figure 5b, we plot the normalized ξ/*d* values for the FCD and Carbopol, to represent the average number of particles participating in nonlocal interactions. We found that the nonlocal interactions span, on average, ∼5–8 particles for both Carbopol (*d*/*w* ≈ 2%) and the FCD measured in either the 500 μm gap CC or the 540 μm capillary (*d*/*w* ≈ 4%). However, as the degree of confinement is increased to ∼8%, as done here for the FCD in the 250 μm capillary, the number of particles participating in the nonlocal interaction decreases to 2. This is in good agreement with our previous work on FCD,^20^ where we varied the degree of confinement, *d*/*w* in the range 5–40% by tuning the crystal aggregate size, *d*. In that study, we observed that the ξ values decreased from spanning ∼10 particles at *d*/*w* = 5% to null under the extreme confinement condition *d*/*w* = 40%.

## Conclusions

We have developed a capillary flow-MRI platform to analyze the flow of complex structured fluids in confined geometries with easily tunable gap size and wall properties. Unlike existing rheo-MRI setups, the platform is easily adaptable to both higher- and lower-field MRI magnets, irrespective of the bore size. The here exploited range of flow confinement sizes, within 100–540 μm, can be expanded down to ∼50 μm and up to cm-scale. By ensuring the mechanical stability of the capillaries under flow, velocities ∼0.01–10 cm/s can be measured with in-plane spatial resolution up to 4.5 × 4.5 μm^2^. The use of cylindrical capillaries enables achieving a high filling factor within the RF saddle coil and acquiring the signal within a slice up to 5 mm thick. As a result, high SNR can be obtained. Notably, the developed flow-MRI platform enables using microfluidics-scale gap sizes, typically used in optical velocimetry, without any requirement of fluids’ optical transparency. Furthermore, MRI velocimetry can uniquely be combined with, e.g., spectroscopy or *T*_1_/*T*_2_ relaxation measurements to probe molecular alignment, particle migration, or phase separation under flow.

The performance of the developed flow-MRI setup was demonstrated here for the study of confinement-induced flow cooperativity for two industrially relevant fluids: Carbopol and FCD. Reproducible 2D velocity maps were obtained with resolution up to 9 × 9 μm^2^ in capillaries with diameters 100–540 μm. Carbopol, with mean particle size ∼2 μm, was found to exhibit pressure-independent cooperativity lengths, ξ, of ∼15 μm in a 100 μm capillary. With this sample, we tackled the quantification of ξ values as low as the MRI resolution. On the other hand, FCD was found to exhibit ξ values in the range 30–150 μm, depending on applied pressure and confinement size. Comparison with ξ values obtained previously for the FCD in a CC geometry,^24^ with a comparable confinement degree but more homogeneous stress distribution, revealed that ξ increases with increasing stress homogeneity within the geometry. Finally, we found that for the FCD, the number of particles participating in the nonlocal interaction decreases from ∼8 to ∼2 particles with the increasing confinement degree from 4 to 8%, in agreement with our previous rheo-MRI findings.^20^

We foresee that the developed capillary flow-MRI platform will aid in achieving, for a wide range of fluid properties, a comprehensive understanding of particle interactions under flow and their dependence on fluid’s microstructure as well as on size, shape, and wall properties, of the confining geometry. The developed knowledge will enable correctly predicting flow profiles, even in industrially relevant conditions, in complex fluids undergoing shear-induced microstructural changes, such as aggregation in protein solutions,^36^ network destruction and rejuvenation in gels^29^ or alignment, crucial in the production of fibers.^37−40^ The flow-MRI platform can be easily adapted to ultrahigh field MRI magnets, boosting the sensitivity of the technique and enabling chemically resolved velocimetric experiments. Such measurements can be exploited in microfluidic studies of the mixing of multiphase fluids or concentration changes during microfiltration. Ongoing and future work will involve monitoring the aforementioned microstructural alterations *in situ* at temperatures beyond the range currently achievable in narrow-gap rheo-MRI CC geometries,^20^ limited at 45 °C by the presence of glued joints. In the future, other RF coil designs, such as striplines,^41^ will be explored for further advancement in sensitivity and resolution, possibly in combination with narrow-bore high- and ultrahigh field MRI magnets.

## Supporting Information Available

Schematic of the rotational rheo-MRI setup, additional experimental results including the μCT results for the determination of the capillary diameters, local flow curves of silicone oil, Carbopol, and FCD, details for determination of the microstructure and global flow behavior of Carbopol and FCD, and ^1^H MRI density profiles measured during flow of FCD. (PDF)
