# Supplementary material for: Capillary Flow-MRI: Quantifying Micron-Scale Cooperativity in Complex Dispersions
Source: Anal Chem. 2023 Oct 5;95(41):15162–70. doi: 10.1021/acs.analchem.3c01108 (PMC10585662; doi:10.1021/acs.analchem.3c01108)
Supplement: Supplementary file 1 — ac3c01108_si_001.pdf [file ac3c01108_si_001.pdf]

Supporting Information

Capillary flow-MRI: quantifying micron-scale cooperativity in complex dispersions

Klaudia W. Milc[1], Thomas Oerther[2], Joshua A. Dijksman[3, 4], John P. M. van Duynhoven[1, 5], Camilla Terenzi[1]

[1]Laboratory of Biophysics, Wageningen University, 6708 WE Wageningen, The Netherlands.

[2]Bruker BioSpin GmbH, 76275 Ettlingen, Germany.

[3]Physical Chemistry and Soft Matter, Wageningen University, 6708 WE Wageningen, The Netherlands.

[4]van der Waals-Zeeman Institute, University of Amsterdam, 1098 XH Amsterdam, The Netherlands.

[5]Unilever Foods Innovation Centre Hive, 6708 WH Wageningen, The Netherlands.

Table of content

Figure S1. a) Scheme of the rotational rheo-MRI setup, with drive shaft length marked as l_{DS}, and the flow geometry fixed in the sensitive region of the probe, marked with a red rectangle. b, c) Commonly used geometries in rotational rheo-MRI, namely CC and CP, with gap size, w. d, e) Illustrative velocity profiles of a Newtonian fluid flowing in a CC and CP respectively.

Figure S2. Diameters of capillaries used in the flow-MRI platform, determined from the intensity profiles vs the position in the x-direction, extracted from 2D axial µCT scans of all capillaries with hydrophilic or hydrophobic walls. The diameters of the capillaries were determined with Avizo software, using the ruler function. The error associated with the measurement is 3.7 µm for all profiles and corresponds to the pixel size.

Figure S3. Local flow curves of silicone oil calculated from the measured velocity profiles (symbols) in capillaries with diameters ranging from 100 to 540 µm, across all tested P_{app} values. The solid line shows the global flow behavior measured with a rheometer.

Figure S4. Micrographs of 0.5% Carbopol and 15% FCD, used in the calculation of the respective autocorrelation functions. The open symbols in the plots represent the radial average of the full 2D function shown in the inset and the solid line is the fit of the exponential decay function described with Eq. 1.

Table S1. Parameters obtained from fitting of the 1D autocorrelation functions of 0.5% Carbopol and 15% FCD with Eq. 1, with their respective fitting errors. Parameter α was set manually and was not a fitting parameter. The characteristic size of the microstructure, d was calculated from r_0.

Figure S5. Global flow curves (open circles), fitted with the Herschel-Bulkley model (solid line) of 0.5% Carbopol, described by the equation of the form $4.07 + 4.4\dot{\gamma}^{0.5}$ and 15% FCD, described by the equation of the form $3.46 + 0.9\dot{\gamma}^{0.7}$.

Figure S6. Global flow curve (solid line) of 0.5% Carbopol compared with the local flow curves calculated from the ^{1}H MRI velocity profiles measured in 540 µm hydrophilic capillary, 100 µm hydrophilic capillary, and 100 µm hydrophobic capillary.

Figure S7. Global flow curve (solid line) of 15% FCD compared with the local flow curves calculated from the ^{1}H MRI velocity profiles measured in 540 µm hydrophilic capillary, and 250 µm hydrophilic capillary.

Figure S8. Radial averages of the 2D density images obtained from the velocimetry measurements of the 15% FCD in a 540 µm capillary and 250 µm capillary under the applied pressures of 0.25 bar, 0.5 bar, 0.75 bar, and 1 bar. Lines of different colors represent the subsequent repeats.

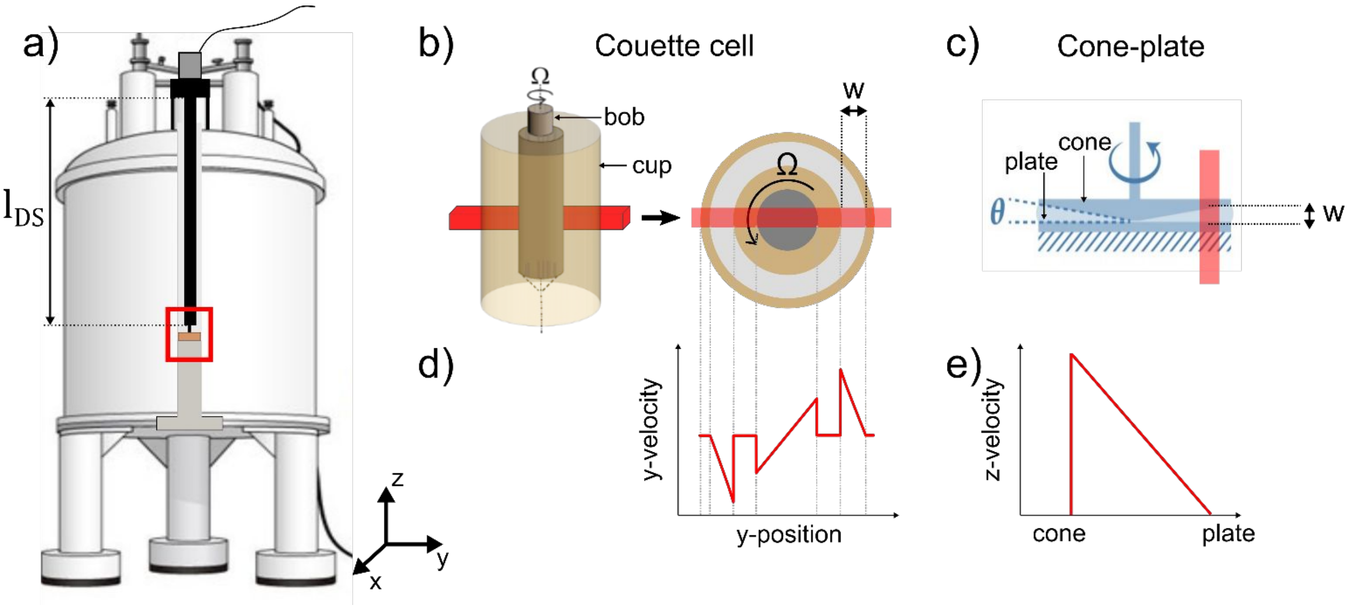

Figure S1. a) Scheme of the rotational rheo-MRI setup, with drive shaft length marked as l_{DS}, and the flow geometry fixed in the sensitive region of the probe, marked with a red rectangle. b, c) Commonly used geometries in rotational rheo-MRI, namely CC and CP, with gap size, w. Red shaded rectangles represent the typical location and orientation of a slice where a 1D velocity profile is measured. d, e) Illustrative velocity profiles of a Newtonian fluid flowing in a CC and CP respectively.

S1. Platform design: radii of capillaries

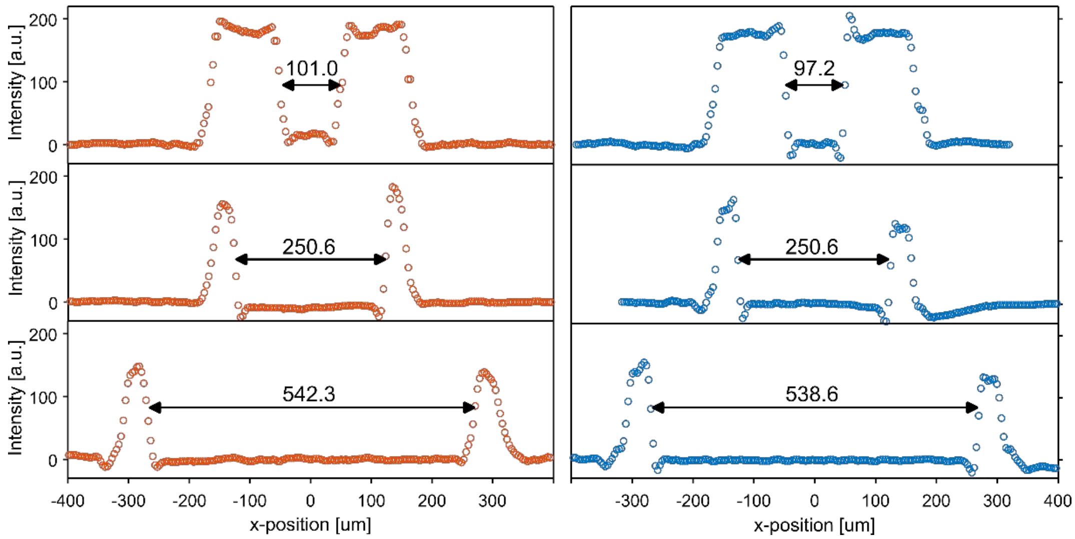

Figure S2. Intensity profiles vs the position in the x-direction, extracted from 2D axial µCT scans of all capillaries with hydrophilic (left column) or hydrophobic walls (right column). The diameters of the capillaries are marked within each profile in units of µm, as measured with Avizo software, using the ruler function. The error associated with the measurement is 3.7 µm for all profiles and corresponds to the pixel size.

S2. Validation of the capillary flow-MRI platform with silicone oil

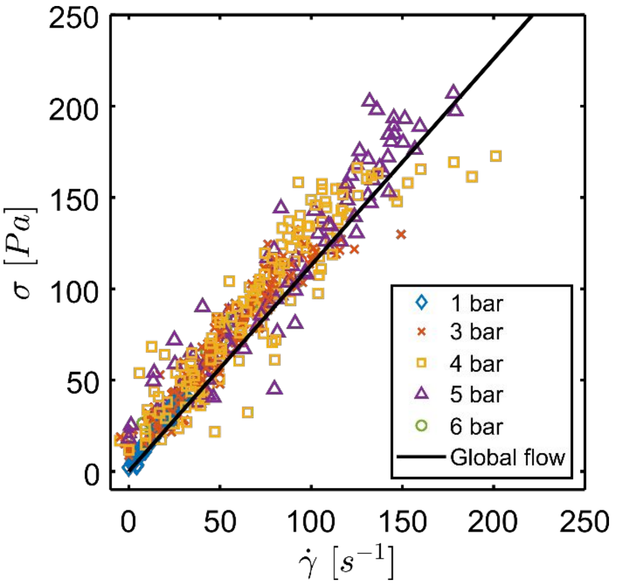

Figure S3. Local flow curves of silicone oil calculated from the measured velocity profiles (symbols) in capillaries with diameters ranging from 100 to 540 µm, across all tested P_{app} values. The solid line shows the global flow behavior measured with a rheometer.

S3. Confined flow of Carbopol and fat crystal dispersion

To establish the necessary condition where the size of the confinement is within 2 orders of magnitude of the microstructure size, we determined the polymer blob size in Carbopol and the crystal aggregate size in the fat dispersion. The micrographs of both samples, together with the associated calculated autocorrelation function, fitted with an exponential decay (Eq. 1) are shown in Fig. S4. The parameters obtained from fitting, together with the calculated sizes of the microstructure are summarized in Table S1.

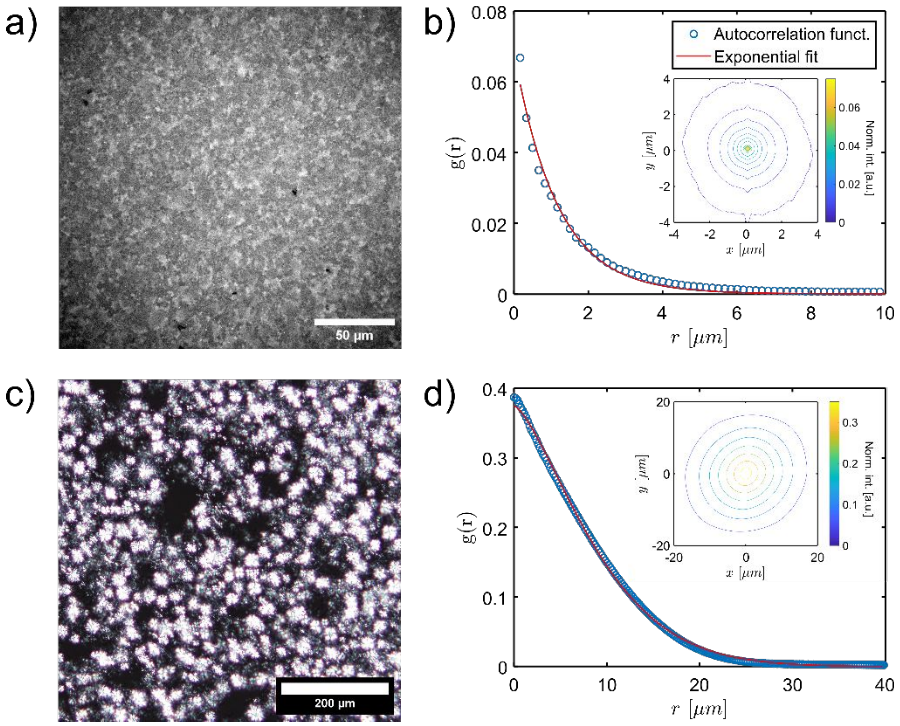

Figure S4. Micrographs of a) 0.5% Carbopol and c) 15% FCD, used in the calculation of the respective autocorrelation functions shown in b) and d). The open symbols in the plots represent the radial average of the full 2D function shown in the inset and the solid line is the fit of the exponential decay function described with Eq. 1.

Table S1. Parameters obtained from fitting of the 1D autocorrelation functions of 0.5% Carbopol and 15% FCD with Eq. 1 with their respective fitting errors. Parameter $\boldsymbol{\alpha}$ was set manually and was not a fitting parameter. The characteristic size of the microstructure, $\boldsymbol{d}$ was calculated from $\boldsymbol{r_0}$.

Sample	g_0	r_0	g_∞	α	$d\ (2 \cdot r_0)$
0.5% Carbopol	0.07 ± 5 x 10^{-4}	1.17 ± 0.01	(1.2 ± 0.3) x 10^{-4}	1.0	2.34 ± 0.02
15% FCD	0.37 ± 4 x 10^{-4}	10.53 ± 0.01	(-3.6 ± 0.4) x 10^{-4}	1.5	21.06 ± 0.02

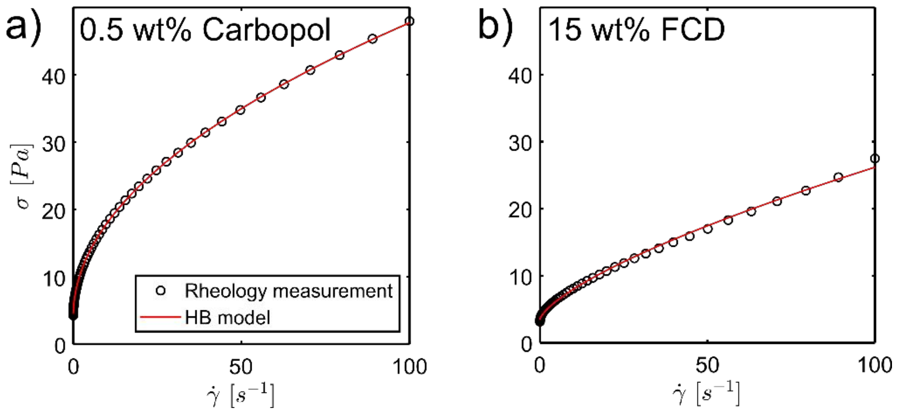

Figure S5. Global flow curves (open circles), fitted with the Herschel-Bulkley model (solid line) of a) 0.5% Carbopol, described by the equation of the form $4.07 + 4.4\dot{\gamma}^{0.5}$ and b) 15% FCD, described by the equation of the form $3.46 + 0.9\dot{\gamma}^{0.7}$.

Figure S6. Global flow curve (solid line) of 0.5% Carbopol compared with the local flow curves calculated from the ^{1}H MRI velocity profiles

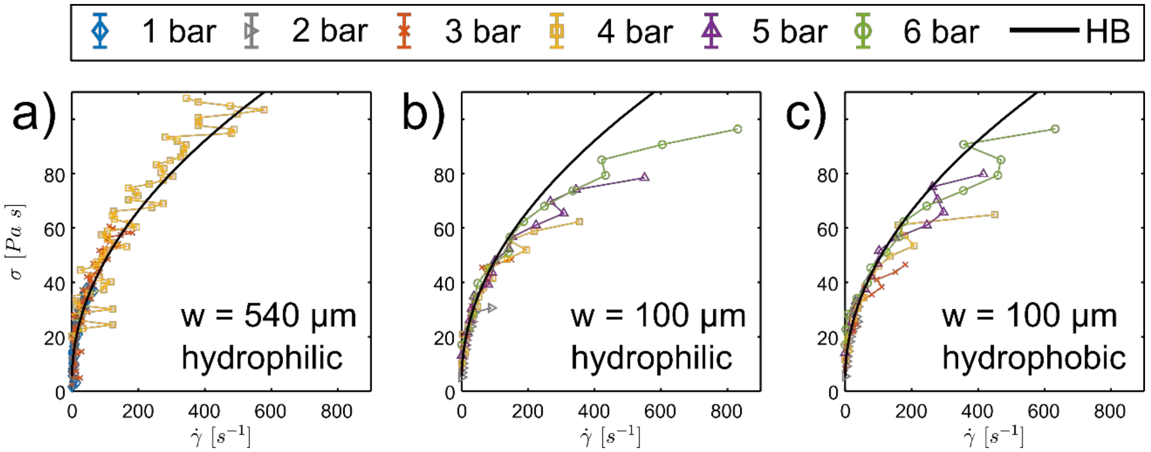

measured in a) 540 µm hydrophilic capillary, b) 100 µm hydrophilic capillary, and c) 100 µm hydrophobic capillary.

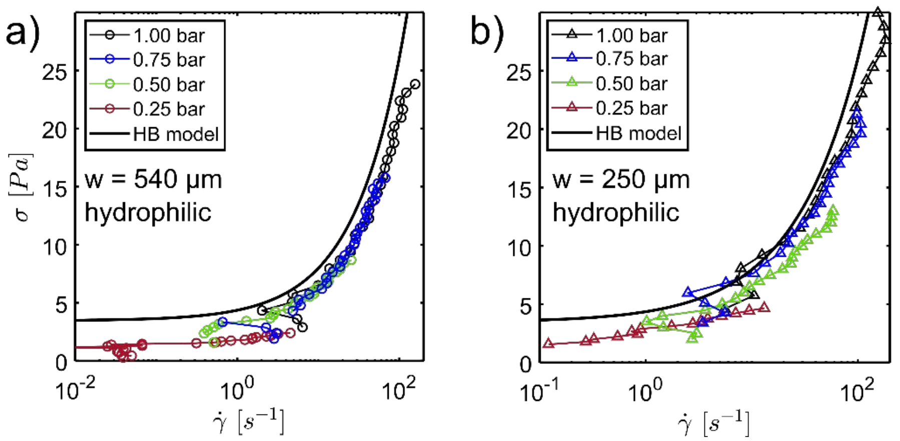

Figure S7. Global flow curve (solid line) of 15% FCD compared with the local flow curves calculated from the ^{1}H MRI velocity profiles measured in a) 540 μm hydrophilic capillary, and b) 250 μm hydrophilic capillary.

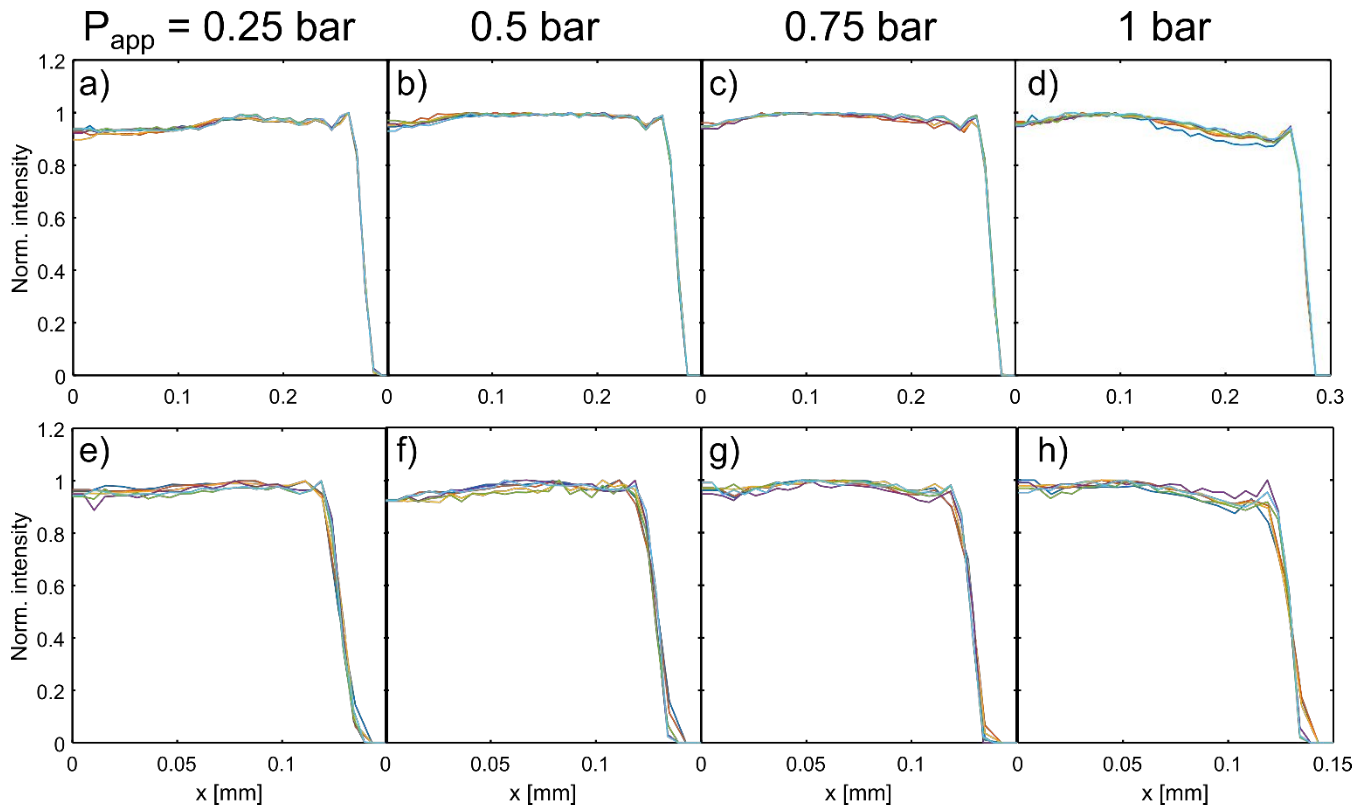

Figure S8. Radial averages of the 2D density images obtained from the velocimetry measurements of the 15% FCD in a 540 μm capillary (top row) and 250 μm capillary (bottom row) under the applied pressures of 0.25 bar (a and e), 0.5 bar (b and f), 0.75 bar (c and g) and 1 bar (d and h). Lines of different colors represent the subsequent repeats.
